# Supplementary material for: The Expanding Phenotype of ZTTK Syndrome Due to the Heterozygous Variant of SON Gene Focusing on Liver Involvement: Patient Report and Literature Review
Source: Genes (Basel). 2023 Mar 17;14(3):739. doi: 10.3390/genes14030739 (PMC10048019; doi:10.3390/genes14030739)
Supplement: Supplementary file 1 [file genes-14-00739-s001.zip › genes-2246700-supplementary.pdf]

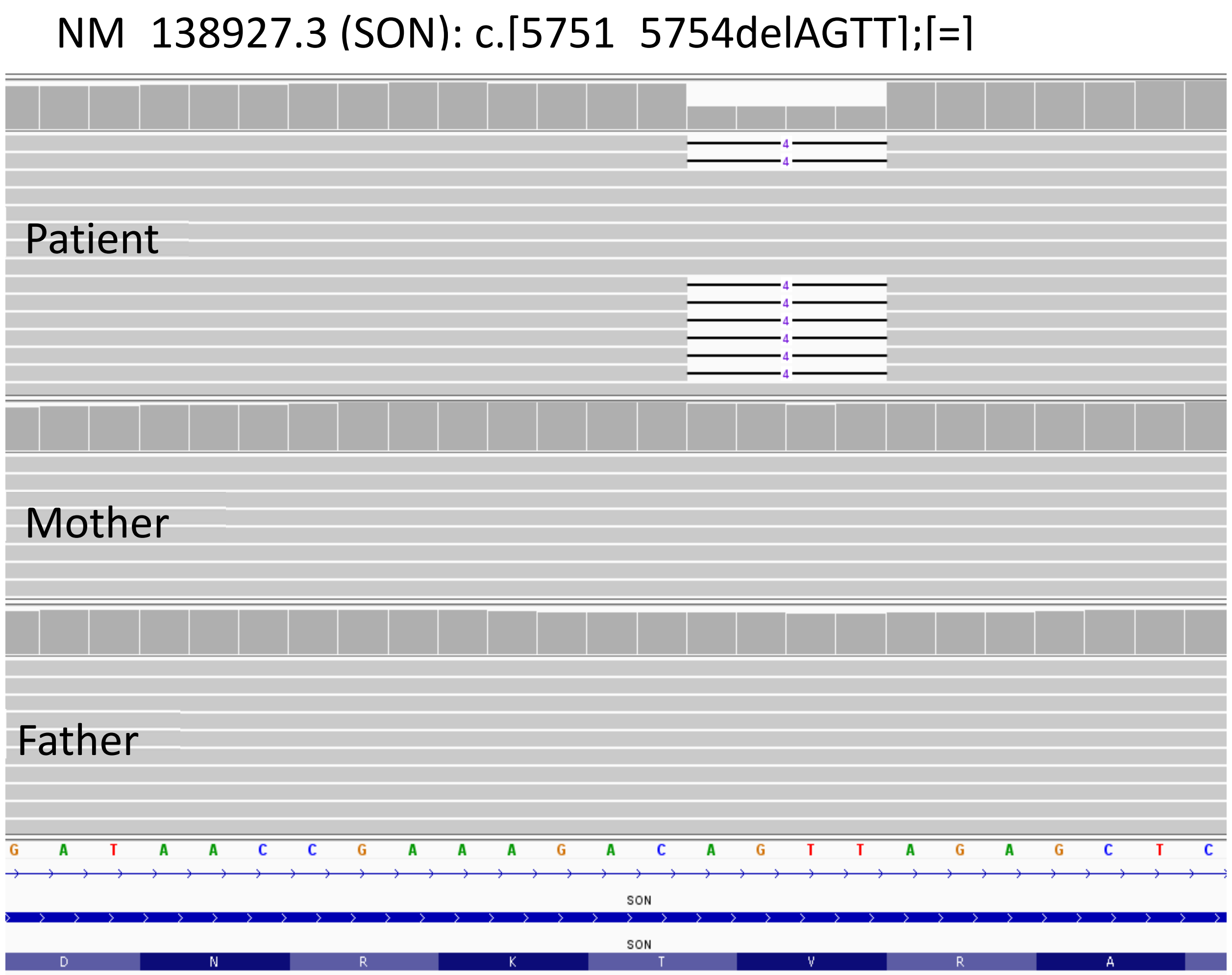

Figure S1. NGS sequencing data from blood samples of the proband and his parents. Identification of *de novo* variant c.5751_5754delAGTT (p.Val1918fsTer87) in SON gene.
